# SAI-YOLO: A Lightweight Network for Real-Time Detection of Driver Mask-Wearing Specification on Resource-Constrained Devices

**DOI:** 10.1155/2021/4529107

**Published:** 2021-11-08

**Authors:** Zuopeng Zhao, Kai Hao, Xiaoping Ma, Xiaofeng Liu, Tianci Zheng, Junjie Xu, Shuya Cui

**Affiliations:** ^1^School of Computer Science and Technology and Mine Digitization Engineering Research Center of Ministry of Education of the People's Republic of China, China University of Mining and Technology, Xuzhou 221116, China; ^2^School of Computer Science and Technology, China University of Mining and Technology, Xuzhou 221116, China

## Abstract

Frequent occurrence and long-term existence of respiratory diseases such as COVID-19 and influenza require bus drivers to wear masks correctly during driving. To quickly detect whether the mask is worn correctly on resource-constrained devices, a lightweight target detection network SAI-YOLO is proposed. Based on YOLOv4-Tiny, the network incorporates the Inception V3 structure, replaces two CSPBlock modules with the RES-SEBlock modules to reduce the number of parameters and computational difficulty, and adds a convolutional block attention module and a squeeze-and-excitation module to extract key feature information. Moreover, a modified ReLU (M-ReLU) activation function is introduced to replace the original Leaky_ReLU function. The experimental results show that SAI-YOLO reduces the number of network parameters and calculation difficulty and improves the detection speed of the network while maintaining certain recognition accuracy. The mean average precision (mAP) for face-mask-wearing detection reaches 86% and the average precision (AP) for mask-wearing normative detection reaches 88%. In the resource-constrained device Raspberry Pi 4B, the average detection time after acceleration is 197 ms, which meets the actual application requirements.

## 1. Introduction

COVID-19 has ravaged the world, resulting in the cumulative global diagnosis of 131.4 million individuals and a cumulative death toll of 2.85 million as of April 6, 2021. Human health was seriously threatened [1]. The COVID-19 virus is transmitted primarily via droplets and close contact [2], and wearing a mask can substantially reduce the rate of the virus spread [3]. In the field of public transportation, because of the confined space of the bus and the high mobility of personnel, bus drivers are required to wear masks throughout the journey. However, to alleviate the discomfort of wearing masks, most drivers often wear masks incorrectly. The incorrect wearing of masks not only fails to protect the drivers but also increases the risk of virus transmission. Thus, it is necessary to detect the normative of wearing a mask.

Currently, the detection algorithm of wearing a mask has been widely studied. Although the traditional machine learning algorithms such as HOG + SVM, HAAR + AdaBoost [4] can detect the mask-wearing specifications, they cannot meet the current requirements because of the general detection effect and long detection time. In 2012, Alex Krizhevsky proposed the deep convolution neural network (CNN) [5] model, AlexNet [6], showing that convolutional neural networks are more efficient in feature extraction than traditional algorithms and have better detection performance. In 2014, Girshick et al. proposed a region-based convolutional neural network (R-CNN) [7], which uses a region-based recognition method to detect objects. In 2015, the Fast R-CNN [8] and Faster R-CNN [9] networks were proposed based on the R-CNN. Both of the above network models belong to a two-stage algorithm. Although they have high detection accuracy, they cannot meet the real-time requirements owing to their complexity and slow detection speed. In 2016, Redmon et al. proposed the “you only look once” (YOLO) [10] algorithm, which combines the phases of candidate selection and object recognition, and used the method to directly predict the boundary box of the target object to improve the detection speed. In the same year, Liu et al. proposed the single shot multibox detector (SSD) [11] algorithm, which draws on the one-stage idea of YOLO and the anchor frame mechanism in the Faster R-CNN. Although detection speed is improved, the accuracy of detection is considerably reduced.

YOLO algorithm has been developed over many years, from YOLOv1, YOLO9000 [12], YOLOv3 [13] to YOLOv4 [14], continuously improving the detection accuracy while maintaining the speed advantage. However, the volume, the number of parameters, and the computing difficulty of the model are still high, making it difficult to apply to embedded devices with limited resources and computing power. Recently, lightweight target detection algorithms have been developed rapidly, such as MobileNet [15], SqueezeNet [16], ShuffleNet [17], and MobileNet-SSD [18], which have good detection performance. In 2020, YOLOv4-Tiny was proposed by AlexeyAB, which maintains a certain detection accuracy while considerably reducing the number of parameters. However, there is still room for improvement in real-time performance. In this study, to further improve the real-time performance of target detection and explore the possibility of model application in embedded devices, a new lightweight target detection network is proposed, which can reduce the size of the model and improve the detection speed while maintaining the recognition accuracy.

The rest of this article is organized as follows.  Section 2 introduces the related research on the detection of face-mask wearing.  Section 3 introduces the network structure of YOLOv4-Tiny and the commonly used attention models.  Section 4 introduces the structure of the new lightweight target detection network SAI-YOLO and describes the innovations and improvements of the network.  Section 5 describes the experimental results and comparative analysis between the proposed network and other networks.  Section 6 concludes this study and provides suggestions for future research directions.

The major contributions of this study are as follows:

1. Propose a new lightweight network SAI-YOLO for driver mask-wearing specification detection based on YOLOv4-Tiny, which has an accuracy of 86.33% and high detection speed on resource-constrained devices
2. Propose two improved structures RES-SEBlock and FPN-CBAM with attention mechanism and experimentally establish that they enhance the model ability to extract key information and reduce the calculation difficulty
3. Collect and label 10,000 images of drivers wearing masks from the real driving environment and establish a dataset called Masked_Imgs which contains three categories of wearing masks, not wearing masks, and not wearing masks correctly
4. The SAI-YOLO network is implemented in the resource-constrained device Raspberry Pi 4B and the collected real images are detected

## 2. Related Works

Face-mask-wearing detection has been extensively studied in recent years, especially after the spread of COVID-19 worldwide. Researchers have focused on mask-wearing detection algorithms based on traditional machine learning and deep learning. Herein, we briefly review the existing studies.

In [19], based on a classical machine learning method, the authors have represented an implementation of principal component analysis (PCA) on masked and nonmasked face recognition. The accuracy of masked face image recognition was on average 72% and nonmasked face recognition was on average 95%, demonstrating that the PCA gives a poor recognition rate for masked face images rather than nonmasked face images. In [20], the authors proposed a hybrid model using ResNet-50 and classical machine learning methods such as SVM, decision tree, and ensemble for face mask detection. Three datasets were used to evaluate the performance of the proposed methodology. The SVM classifier achieved an accuracy of 99.64% in the real-world masked face dataset and 99.49% in the simulated masked face dataset. The authors in [21] also used ResNet-50. They proposed another model by using the YOLOv2 for the detection of medical face masks instead of the SVM. Two medical face mask datasets were combined as one dataset for training and testing. The proposed model achieved the highest average precision percentage of 81% when using Adam optimizer. In [22], the authors proposed a system using LogitBoost for detecting the presence or absence of the mandatory medical mask in the operating room. In order to have as few false positive face detections as possible without losing mask detection, they used two face detectors, one for detecting faces and the other for detecting medical masks. The proposed system rendered a recall above 95% with a false positive rate below 5% for detecting faces and surgical masks. In [23], the authors built a dataset called Indian facemasks detection dataset [24] centered on the Indian community where, alongside standard surgical masks, images of people wearing other types of home-made veils like dupattas and handkerchiefs were also included. Based on testing on the established dataset, they concluded that YOLOv4 transcended both YOLOv3 and SSD-MobileNet V2 in sensitivity and precision.

However, the above-mentioned studies only focused on whether people wear masks, but ignored whether people wear masks correctly. Although researchers and scientists have shown that wearing a mask can help reduce the spread of COVID-19, incorrect way of wearing masks does not ensure the same. At present, only a small number of researchers pay attention to whether people wear masks correctly and have achieved good results. In [25], the authors developed a new facemask-wearing condition identification method by combining image super-resolution and classification network (SRCNet). They regarded facemask-wearing condition identification as a kind of three-category classification problem, including no facemask-wearing, incorrect facemask-wearing, and correct facemask-wearing. The SRCNet achieved 98.70% accuracy and outperformed traditional end-to-end image classification methods.

In general, face mask wearing detection runs on a high-performance computing platform. However, in some special scenarios such as buses, it needs to run on resource-constrained devices. There are no lightweight models that can be used on resource-constrained devices for real-time detection of facemask-wearing condition. Hence, the SAI-YOLO is proposed to identify facemask-wearing condition, which is expected to have an application value, especially in the prevention of an epidemic such as COVID-19.

## 3. Methodology

### 3.1. YOLOv4-Tiny Lightweight Network

**Figure 1:**
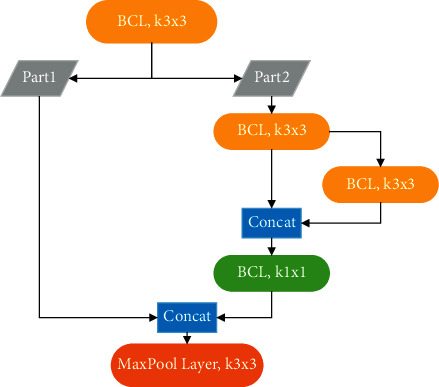
CSPBlock structure.

**Figure 2:**
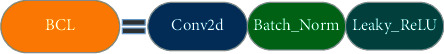
Structure of base convolutional layer.

YOLOv4-Tiny is a simplified version of YOLOv4, which greatly reduces the number and volume of parameters while maintaining high recognition accuracy. Compared with YOLOv4, the backbone network of YOLOv4-Tiny consists of only three base convolution layers (BCLs) and three CSPBlocks.  Figure 1 shows the structure of the CSPBlock. Each CSPBlock comprises three base convolution layers of size 3 × 3 and one of size 1 × 1. Moreover, two residual edges are added to improve the learning performance of the network, and the maximum pooled layer with a convolution core size of 2 × 2 is added as output. The CSPBlock incorporates the CSPNet [26] structure, doubling the number of gradient paths by using a split-merge strategy across phases, thereby effectively reducing the computational load of the model. In addition, due to the cross-stage strategy, the adverse effects of cascading with explicit feature mapping replicas can be mitigated. As shown in Figure 2, the base convolution layer includes the convolution layer with a core size of 3 × 3, the normalization layer, and the Leaky-ReLU used as the activation function.

**Figure 3:**
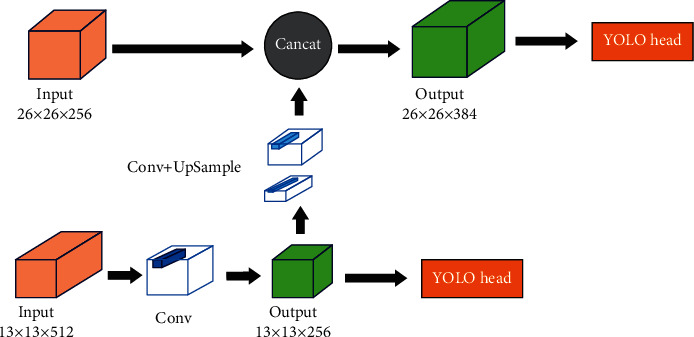
Neck structure.

The backbone network of YOLOv4-Tiny is shown in Table 1, where C represents the number of channels output and K represents the size of the convolution core. Since several convolution cores of different sizes are used in the CSPBlock module, they are listed separately in the table and S represent the step stride. In the middle of the backbone network and the head, a neck is added, which uses the FPN [27] structure for reference to collect and fuse the two effective feature layers output by the second CSPBlock module and the last convolution layer. In the neck, the output of the last convolution layer is operated on two operations: the first is to output the predicted result directly after convolution, and the second is to do convolution and upsampling, and stack the results with the CSPBlock output. Compared with the PANet [28] structure used by YOLOv4, the FPN used by the neck improves the path from bottom to top, transfers the strong features of the lower layer to the higher layer, and improves the network's ability to extract information from the lower layer and the accuracy of prediction. The neck structure is shown in Figure 3.

### 3.2. Attention Mechanism

The attention mechanism derives from the selective attention mechanism of the human eye, which allows the eye to filter out a large amount of useless information and focus only on a specific object. Attention models were originally applied to the recurrent neural network (RNN) and have since been widely used in CNN. The attention mechanism trains an additional mask layer to acquire the importance of different channels in the attention domain and assigns weight values corresponding to the importance to enable the feature extractor pay more attention to important information. Both the squeeze-excitation method proposed by Hu et al. [29] and the residual attention network proposed by Wang et al. [30] apply the attention mechanism and achieve high-accuracy detection results.

**Figure 4:**
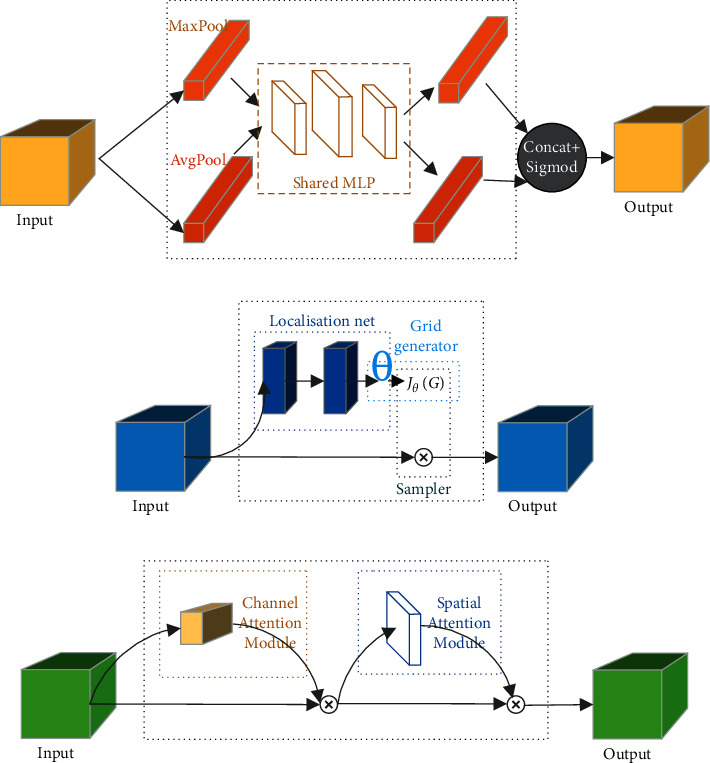
Attention module. (a) Channel attention. (b) Spatial attention. (c) Attention module.

Early attention mechanism studies analyzed the brain imaging mechanism and used a winner-take-all [31] mechanism to study how to model attention. In recent years, most research on the combination of deep learning and visual attention mechanisms has focused on the use of masks to form attention mechanism. The principle of mask is to identify the key features in the image data through additional layers of new weight. Through learning and training, the deep neural network can learn the areas that need attention in each new image, which forms attention. Now, in the field of computer vision, the domains of attention can be divided into spatial, channel, and mixed domains. Among them, the channel attention module is mainly modeled by the correlation between different channels and key information, and the original feature layer is weighted to highlight the channels with key information and suppress the rest of the channels. The structure of the channel attention module is shown in Figure 4(a).

The spatial attention module proposed by Jaderberg et al. [32] forms a mask layer with the degree of pixel correlation by extracting the relationship and relative position of each pixel point in the space. By calculating the weights of the pixel points through the degree of correlation, the pixel points carrying important information are retained to achieve the function of feature selection and fusion. The structure of the spatial attention module is shown in Figure 4(b).

Because the spatial attention module ignores the information in the channel domain and processes the image features in the channel equally, it can only be applied in the original image feature extraction stage. By contrast, the channel attention module focuses only on the global average pooling of information within a channel, ignoring local information within each channel. Because of the limitations of a single attention module, researchers have begun to mix different attention modules, and many excellent methods have been proposed. In [33], the authors proposed DANet, which combines the spatial attention module and channel attention module in parallel to form a hybrid domain attention mechanism. In [34], the authors proposed a convolutional block attention module (CBAM), which combines the channel attention module and the spatial attention module in series to form a hybrid domain attention mechanism. They also found through experiments that placing the channel attention module first can achieve better results. The structure of the CBAM is shown in Figure 4(c).

## 4. Proposed Network SAI-YOLO

To meet the requirements of target detection on resource-constrained embedded devices and further improve the recognition speed, SAI-YOLO is proposed. It improves the backbone and feature pyramid of YOLOv4-Tiny, which in turn improves the real-time detection performance of the network while maintaining the recognition accuracy.

**Figure 5:**
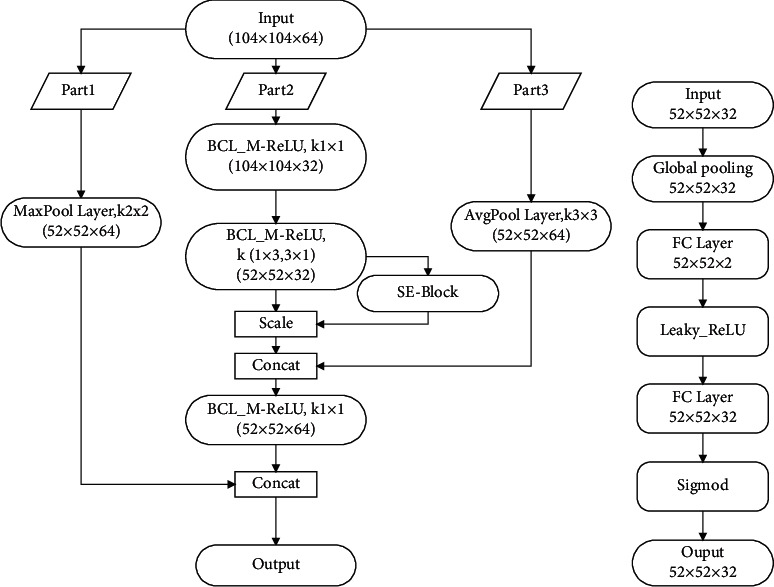
Structure of RES-SEBlock. (a) RES-SEBlock. (b) SE-Block.

First, YOLOv4-Tiny uses three CSPBlock modules in the backbone. Although the CSPBlock module reduces the calculation difficulty and the number of parameters of the network, the real-time detection performance still needs to be improved. To improve the detection speed, this study uses two RES-SEBlock modules to replace two CSPBlock modules. The structure of RES-SEBlock is shown in Figure 5(a). In the RES-SEBlock module, referring to the intensely inverted residual network [35], the input is processed along three paths. In path 1, a maximum pooling layer with a convolution kernel size of 2 × 2 is added to the residual edge. In path 2, the original 3 × 3 convolution kernel is replaced by 3 × 1 and 1 × 3 convolution kernels based on the Inception [36] structure. This not only reduces the parameters of the module but also expands the receptive field of feature extraction. In addition, in the convolution process, to improve the ability to extract key features, the SE-Block module is added. The structure of the SE-Block module is shown in Figure 5(b). SE-Block combines the channel attention mechanism and assigns different weights to different features, which enables the network to extract key features more effectively. Compared with the CSPBlock module, the RES-SEBlock module greatly reduces the number of network parameters and the difficulty of calculation, which is more conducive to the improvement of real-time detection performance. In path 3, referring to the ShuffleNet model, an average pooling layer with a convolution kernel size of 3 × 3 is added to the residual edge. After the average pooling layer, the output is added to path 2 as an additional residual connection.

To accurately measure the difference in computational difficulty between the two modules, a comparison is made in terms of the floating-point operator (FLOPs). When the input size is *H*_in_ × *W*_in_ × *C*_in_ and the output is *H*_out_ × *W*_out_ × *C*_out_, the convolution kernel size is set to *k*, the convolution kernel parameter is set to *k*^2^, and the FLOPs is calculated as follows:

(1) $$\begin{array}{c} \text{FLOPs}=H_{\text{out}}\times W_{\text{out}}\times {\text{C}}_{\text{in}}\times C_{\text{out}}\times k^{2}. \end{array}$$

We assume that the original image size entered by the backbone network is 416 × 416, and after two convolutions, the output size is 104 × 104, and the number of channels is 64. Based on the above data, the calculated FLOPs for CSPBlock are

(2) $$\begin{array}{c} \text{FLOPs}=104^{2}\times 3^{2}\times 64^{2}+104^{2}\times 3^{2}\times 32\times 64+104^{2}\times 3^{2}\times 32^{2}+104^{2}\times 64^{2}=7.42\times 10^{8}. \end{array}$$

The FLOPs of RES-SEBlock are

(3) $$\begin{array}{c} \text{FLOPs}=104^{2}\times 1^{2}\times 64\times 32+52^{2}\times 3^{2}\times 32^{2}\times 1^{2}+52^{2}\times 96\times 64\times 1^{2}+64\times 52^{2}\times 2^{3}+52^{2}\times \left(32+2+2\right)=6.51\times 10^{7}. \end{array}$$

Formulas (2) and (3) show that the FLOPs of the RES-SEBlock module are about one-eleventh of the CSPBlock module, which also shows that the RES-SEBlock module can improve the speed of network detection.

Although RES-SEBlock accelerates network detection, compared with CSPBlock, its ability to extract features and the accuracy of recognition decrease. To solve this problem, two improvement measures are adopted. First, a CBAM structure is added to the feature pyramid, which forms the FPN-CBAM structure as shown in Figure 4(c). The CBAM combines the spatial attention module with the channel attention module, wherein the former is used to analyze the dependence of features in space and the latter is used to weigh the feature maps of different channels. Thus, more favorable feature maps are obtained for classification. After the CBAM module, invalid features are suppressed, while key features are extracted and improved. The resulting feature map is more conducive to target classification and detection, thus improving the accuracy of network detection. Second, the activation function Leaky-ReLU is replaced by M-ReLU [37]. Leaky_ReLU and ReLU are not differentiable near 0, creating problems for this gradient descent process. By slightly modifying the ReLU, M-ReLU makes it differentiable for all the weight values. M-ReLU is defined by

(4) $$\begin{array}{c} \sigma ={\left(\max0,z\right)}^{1.0000001}. \end{array}$$

**Figure 6:**
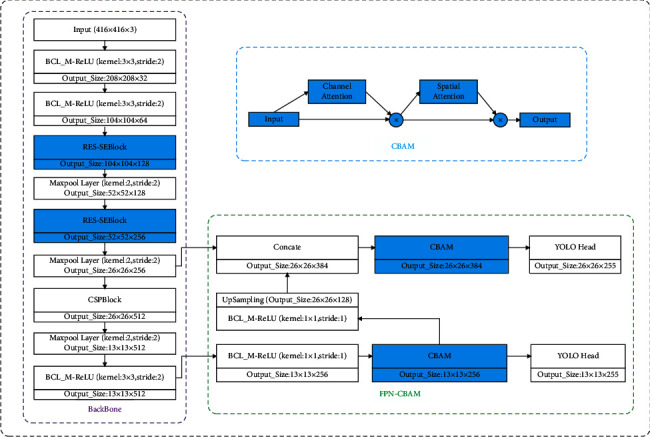
The proposed SAI-YOLO network structure.

Based on the above improvements, the overall structure of SAI-YOLO is shown in Figure 6. In the SAI-YOLO network, two RES-SEBlock modules are mainly used to replace two CSPBlock modules, leaving only one CSPBlock module to extend the feature extraction field. In addition, the use of the FPN-CBAM module instead of the FPN module in YOLOv4-Tiny can increase the extraction of key feature information and inhibit the extraction of unnecessary information.

## 5. Experimental Results and Analysis

### 5.1. Dataset and Experimental Settings

In this study, the experimental platform is Intel Core I5 9400F processor, with NVIDIA GeForce RTX 2070S 8 G memory, and the software environment is PyCharm2020.1.3, with PyTorch deep learning framework. The VOC2007 and VOC2012 datasets [38], and MAFA datasets [39] are used to pretrain models. Finally, the Masked_Imgs dataset collected from the driver video monitoring platform is used to train the network and test the performance of SAI-YOLO and other popular target detection networks.

VOC2007 and VOC2012 datasets are divided into 20 target object categories (without background). This study uses the VOC07 + 12 combination to train the network, which consists of 16,551 images, including the VOC2007 training set and VOC2012 training and test set. The network performance was tested using the VOC2007 test set containing 4,952 images.

The MAFA dataset, published in 2017 by Ge et al., consists of 30,811 images and contains 35,806 obscured faces. Of the 30,811 tagged images, 25,876 were for the training set and 4,935 were for the test set. The labels in the training set are divided into three categories: mask face, 2eyes, and occluder. The test set is also labeled the same, replacing the original label types.

**Figure 7:**
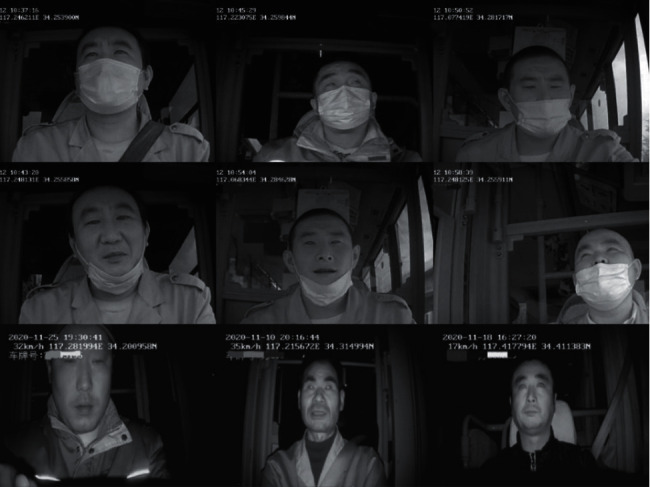
Masked_Imgs dataset.

All images in the Masked_Imgs dataset are provided by the driver's video monitoring platform. A total of 600 driving videos of different drivers are provided by the monitoring platform, including 200 each in the morning, at noon, and in the evening. In each video, five groups of images are collected. Each group contains three types of images: the driver does not wear a mask, correctly wears a mask, and incorrectly wears a mask. In addition, to enhance the diversity of the dataset, 1,000 images are collected from these videos under special conditions such as strong light exposure and face obstacle occlusion. A total of 10,000 images are captured in this study, as shown in Figure 7. These images are divided into training and verification sets in a 9 : 1 ratio. Finally, 9,000 images of the training set and 1,000 images of the verification set are obtained. LabelImg [40] is used to label the images of the dataset and generate the corresponding XML file.

### 5.2. Performance Evaluation Indicators

There are many evaluation indexes for the object detection algorithm [41], such as the commonly used mAP, FLOPs, and MB. In this study, accuracy, precision, recall rate, and mAP are used to evaluate the performance of the target detection model on the dataset. Accuracy is the ratio of correctly predicted observations. The recall rate is the ratio of the number of samples that are correctly predicted for the class to the total number of samples; it is also called the sensitivity or hit rate. Precision refers to the ratio of the number of category samples correctly predicted to the total number of samples predicted for that category [42]. The calculation methods for these indicators are as follows:

(5) $$\begin{array}{c} \text{accuracy}=\left(1-\frac{a}{m}\right)\times 100\%,\\ \text{precision}=\frac{\text{TP}}{\text{TP}+\text{FP}}\times 100\%,\\ \text{recall}=\frac{\text{TP}}{\text{TP}+\text{FN}}\times 100\%, \end{array}$$

where *a* is the number of samples misclassified, *m* is the total number of samples, TP is the number of positive samples correctly classified by the model, and FP is the number of negative samples misclassified by the model as positive samples. FPS is used to assess the real-time detection performance of the model, and MB is used to assess the size of the model. By weighing these performance indicators experimentally, a more applicable model for embedded mobile devices is discussed.

### 5.3. Experimental Results and Analysis

#### 5.3.1. Model Performance Comparison

In this section, experiments were performed on VOC, MAFA, and Masked_Imgs datasets using different popular lightweight object detection networks, namely, MobileNet-SSD, YOLOv3-Tiny, YOLOv4-Tiny, and the proposed SAI-YOLO.

**Figure 8:**
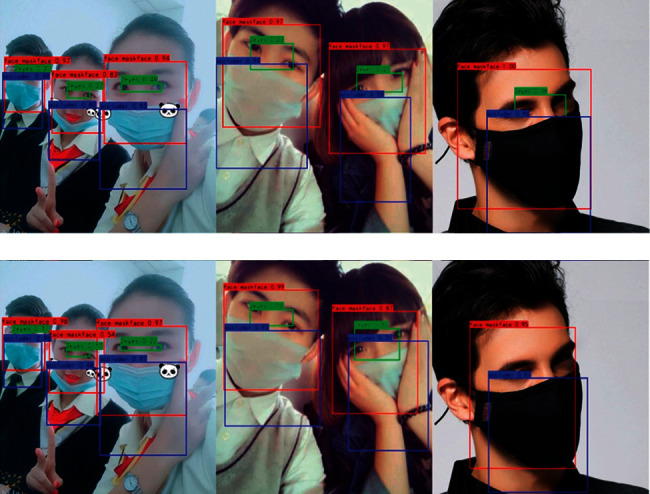
Effect comparisons on MAFA dataset: (a) SAI-YOLO and (b) YOLOv4-Tiny.

To ensure the fairness of the results, the experiments were tested using a weight file with the highest mAP value. The experimental results on the VOC and MAFA datasets are shown in Tables 2 and 3. In terms of detection accuracy, as shown in Table 2, YOLOv3-Tiny achieved the minimum mAP value of 70.87%, followed by MobileNet-SSD, with a mAP of 72.70%. The second highest mAP was the YOLOv4-Tiny network, which was 75.67%. The SAI-YOLO network was 1.92% higher than YOLOv4-Tiny and much higher than the other two networks. In Table 3, it can be seen that the mAP values tested on the MAFA dataset were lower than those on VOC dataset. However, the SAI-YOLO network still achieved the maximum mAP value of 68.53%, 6.18% higher than the minimum mAP value achieved by YOLOv3-Tiny at the cost of less model size. Compared to the tests on the VOC0712 and MAFA datasets, the mAP values tested on the Masked_Imgs dataset have considerably increased. As shown in Table 4, the SAI-YOLO network achieved a maximum mAP value of 86.33%, 0.87% higher than that of YOLOv4-Tiny and 2.79% higher than that of MobileNet-SSD. YOLOv3-Tiny achieved a minimum mAP value of 80.14%. In addition, in the three experiments, the SAI-YOLO network model size was the smallest, which confirms that the designed network can be more easily applied to resource-constrained devices. The results of SAI-YOLO and YOLOv4-Tiny networks on the MAFA dataset are shown in Figure 8. It can be seen that both networks have poor detection performance for eyes, but SAI-YOLO is generally better for eye detection. This also indicates that the proposed network enlarges the eye feature weight and increases the extraction of eye features.

**Figure 9:**
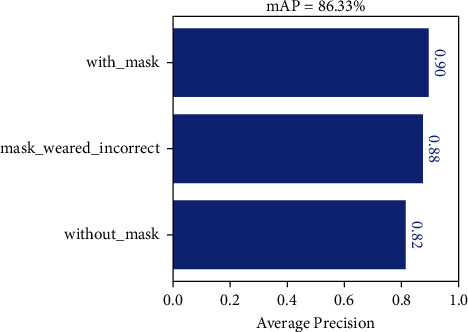
Average precision of the SAI-YOLO in Masked_Imgs dataset.

**Figure 10:**
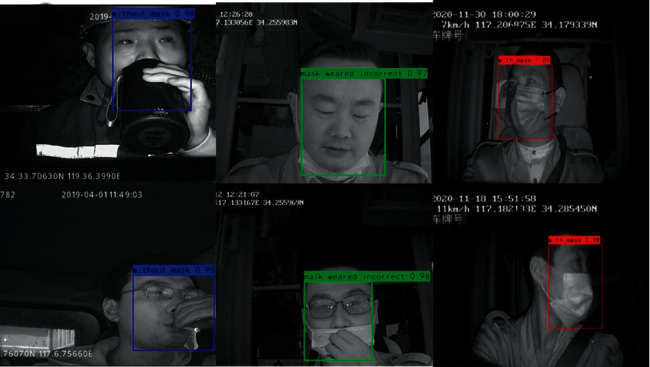
Detection results on Masked_Imgs.

When testing on the Masked_Imgs dataset using the SAI-YOLO network, the AP values for each category are shown in Figure 9. Here, the recognition AP value was 0.9 for correctly wearing a mask and 0.88 for incorrectly wearing a mask. The lowest AP value was identified for wearing a mask, with only 0.82. This is because, in the actual driving environment, drivers may change their head posture for various purposes, including drinking water, smoking, and watching mobile phones, which covers their faces [43, 44].  Figure 10 shows the effect of SAI-YOLO network prediction on the Masked_Imgs dataset.

#### 5.3.2. FPS Comparison and Ablation Experiment

To verify the real-time detection performance of SAI-YOLO, different resolution sizes of video are input into SAI-YOLO and YOLOv4-Tiny. The test was conducted on NVIDIA GeForce RTX 2070S. As shown in Table 5, FPS gradually increased as the resolution of the input video decreased. Compared with YOLOv4-Tiny, the proposed networks had higher FPS values. When the video resolution was 640×480, the FPS reached 174.2. This also proves that the SAI-YOLO network has high detection speed and real-time performance.

In addition, to verify the effect of the designed modules on the network performance, RES-SEBlock, FPN-CBAM, and M-ReLU activation function were added to the YOLOv4-Tiny model separately and tested while keeping the other experimental parameters unchanged. The dataset used in the experiment was VOC2007. By comparing the first and fourth rows of Table 6, it can be seen that only using RES-SEBlock to replace CSPBlock not only reduced the model size but also improved the detection speed; the FPS increased by 24.9. However, as the speed increased, the accuracy decreased by 3.97%. Comparison of the second and fourth rows indicates that using the FPN-CBAM module increased the accuracy by 4.2%; however, because of the increase in the parameters, the model size increased slightly and the FPS decreased by 7.8. By comparing the results of the third and fourth lines, it can be seen that with the use of M-ReLU as activation function, the detection accuracy achieves 78.54%, 2.87% higher than the YOLOv4-Tiny.

#### 5.3.3. Illumination and Occlusion Experiment

**Figure 11:**
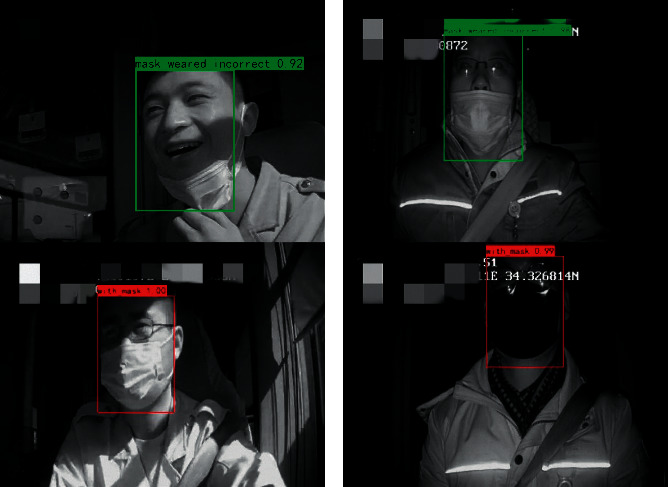
Illumination test. (a) Strong light exposure and (b) poor light exposure.

**Figure 12:**
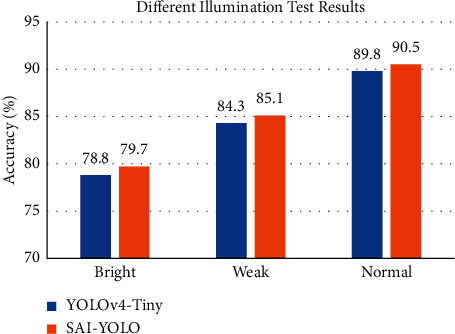
Different illumination test results.

**Figure 13:**
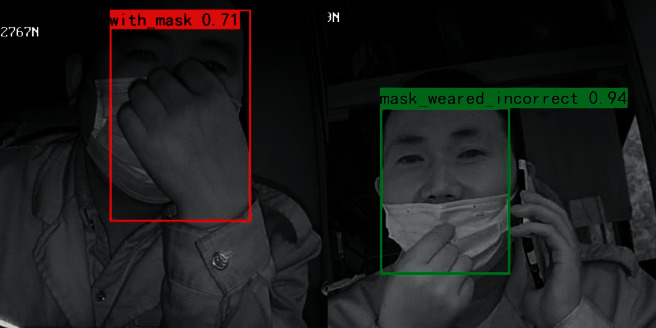
Occlusion test.

In this section, we tested the performance of the SAI-YOLO and YOLOv4-Tiny models by running them on a set of images containing specific characteristics types, including occlusion, poor lighting, and intense lighting. In the illumination test, 450 images were used; 150 in intense light, 150 in weak light, and 150 in normal light. Some of the renderings are shown in Figure 11, where (a) is the intense-light detection result diagram and (b) is the weak-light detection result diagram. The accuracy under three different lighting conditions is shown in Figure 12. It can be seen that the SAI-YOLO network showed higher detection accuracy than YOLOv4-Tiny in intense, weak, or normal light conditions. In the obstacle-blocking test, the detection effects of some images were obtained, as shown in Figure 13, and the statistical results are listed in Table 7. The detection accuracy of the SAI-YOLO network under partial occlusion was 71.5%, which is 2.1% higher than that of YOLOv4-Tiny. Overall, the proposed SAI-YOLO model can complete real-time detection of targets with high accuracy and overcome the effect of light and obstacles; moreover, it has superior performance compared to YOLOv4-Tiny.

#### 5.3.4. Experiments in Raspberry Pi 4B

In the above experiments, the device used is NVIDIA GeForce RTX 2070S, and experiments have verified that the SAI-YOLO network has a faster detection speed than other lightweight networks. In this section, the SAI-YOLO network and two other lightweight networks are ported to the resource-constrained device Raspberry Pi 4B for testing. The dataset used for the test is Masked_Imgs.

**Figure 14:**
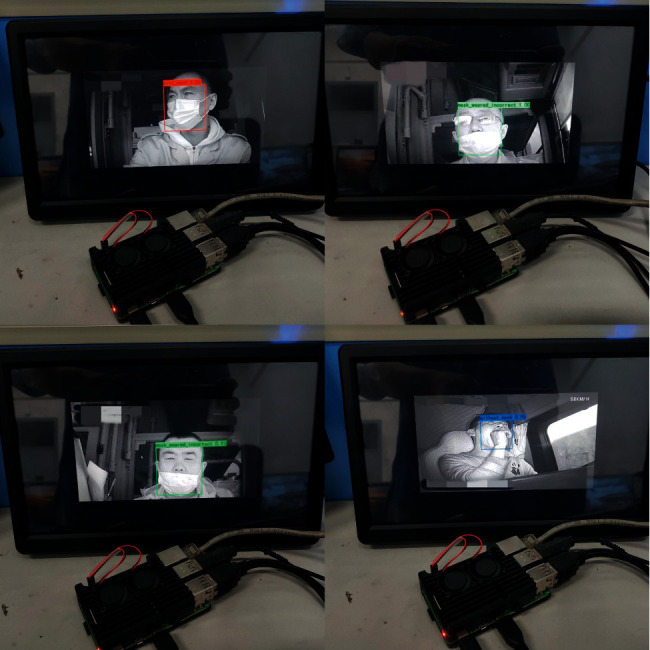
Detection test on Raspberry Pi 4B.

This experiment mainly tested the average detection time of a single image. To increase the speed of the detection performance on Raspberry Pi 4B, we used ncnn [45], a high-performance neural network inference computing framework optimized for mobile platforms. As shown in Table 8, the average detection time of the SAI-YOLO network was the shortest, at 197 ms, which is 45 ms lower than that of the YOLOv4-Tiny network and 24 ms lower than that of the MobileNet-SSD network. This also verifies the superiority of the SAI-YOLO network in terms of detection speed.  Figure 14 shows the effect of the SAI-YOLO network on drivers wearing mask recognition on the resource-constrained device. In summary, compared with YOLOv4-Tiny, the SAI-YOLO network reduces the parameter amount and calculation difficulty, improves the detection speed, guarantees certain recognition accuracy, and is more suitable for porting to resource-constrained devices.

## 6. Conclusions

To solve the problem of detecting drivers wearing masks in the epidemic period, a lightweight network SAI-YOLO based on YOLOv4-Tiny was proposed. The network replaces two CSPBlock structures with RES-SEBlock structures, which reduces the number of network parameters and computational difficulties. At the same time, a hybrid attention mechanism is added to improve the extraction of key feature information. The SAI-YOLO network can detect the wearing specifications of face masks in real-time and efficiently on resource-constrained devices. Using MAFA, VOC2007, VOC2012, and Masked_Imgs dataset to train and test the network, the experimental results showed that the SAI-YOLO network achieved a mAP of 86% for face-mask-wearing detection and recognition of 88% for mask-wearing specifications. The average detection time after acceleration on the resource-constrained device Raspberry Pi 4B was 197 ms, which meets the practical application requirements.

In the future, we will continue to optimize the SAI-YOLO network structure, reduce the number of network parameters and computing difficulties, and conduct more experiments in embedded devices. In addition, the network has a high rate of error detection for wearing mask specifications under abnormal angles, which can be improved in the future.

## Figures and Tables

**Table 1:** Structure of YOLOv4-Tiny.

Input	Operator	c	k	s
416 × 416 × 3	Base Conv2d	32	2	2
208 × 208 × 32	Base Conv2d	64	2	2
104 × 104 × 64	CSPBlock	128	3,1	1
52 × 52 × 128	CSPBlock	256	3,1	1
26 × 26 × 256	CSPBlock	512	3,1	1
13 × 13 × 512	Base Conv2d	512	3	1

**Table 2:** Results on the VOC0712 dataset.

Network	Dataset	Model size (MB)	mAP (%)
MobileNet-SSD	VOC0712	23.3	72.70
YOLOv3-Tiny	VOC0712	33.1	70.87
YOLOv4-Tiny	VOC0712	22.6	75.67
SAI-YOLO	VOC0712	20.4	77.59

**Table 3:** Results on the MAFA dataset.

Network	Dataset	Model size (MB)	mAP (%)
MobileNet-SSD	MAFA	24.5	64.97
YOLOv3-Tiny	MAFA	34.6	62.35
YOLOv4-Tiny	MAFA	23.2	66.94
SAI-YOLO	MAFA	21.4	68.53

**Table 4:** Results on the Masked_Imgs dataset.

Network	Dataset	Model size (MB)	mAP (%)
MobileNet-SSD	Masked_Imgs	23.1	83.54
YOLOv3-Tiny	Masked_Imgs	31.6	80.14
YOLOv4-Tiny	Masked_Imgs	22.4	85.46
SAI-YOLO	Masked_Imgs	20.1	86.33

**Table 5:** FPS of the networks in videos of different resolutions.

Video resolution	YOLOv4-Tiny	SAI-YOLO
1920 × 1080	101.5	114.5
800 × 600	125.4	144.3
640 × 480	157.7	174.2

**Table 6:** The impact of different modules on the network.

Network	Dataset	Model size (MB)	mAP (%)	FPS
YOLOv4-Tiny (RES-SEBlock)	VOC0712	19.5	72.70	150.3
YOLOv4-Tiny (FPN-CBAM)	VOC0712	23.2	80.87	117.6
YOLOv4-Tiny(M-ReLU)	VOC0712	22.6	79.54	124.8
YOLOv4-Tiny	VOC0712	22.6	76.67	125.4

**Table 7:** Results of obstruction detection.

Network	Accuracy (%)	Number of test images
YOLOv4-Tiny	71.5	200
SAI-YOLO	73.6	200

**Table 8:** The average time of different networks on Raspberry Pi 4B.

Network	Average time (ms)
MobileNet-SSD	1368
YOLOv4-Tiny	1537
SAI-YOLO	1194
MobileNet-SSD(ncnn)	221
YOLOv4-Tiny(ncnn)	242
SAI-YOLO(ncnn)	197

## Data Availability

The data used to support the findings of this study cannot be shared at this time as the data also form part of an ongoing study.

## Abbreviations

HOG: Histogram of oriented gradients
HAAR: Haar-like features
AdaBoost: Adaptive Boosting
SVM: Support vector machines
ReLU: Rectified linear unit
mAP: Mean Average Precision
AP: Average Precision
CSPBlock: Cross stage partial block
RES-SEBlock: Residual and squeeze-excitation block
FPN-CBAM: Feature pyramid network with convolutional block attention module
YOLO: You only look once
SSD: Single shot multibox detector
CNN: Convolution neural network
PCA: Principal component analysis
LogitBoost: Logistic Boosting
SRCNet: Super-resolution and classification network
FPN: Feature pyramid network
BCL: Base convolution layer.
